# Comparison of AlN vs. SIO_2_/LiNbO_3_ Membranes as Sensitive Elements for the SAW-Based Acceleration Measurement: Overcoming the Anisotropy Effects

**DOI:** 10.3390/s20020464

**Published:** 2020-01-14

**Authors:** Sergey Yu. Shevchenko, Denis A. Mikhailenko, Oleg A. Markelov

**Affiliations:** Department of Laser Measuring and Navigation Systems, Saint Petersburg Electrotechnical University, 5 Prof. Popov Str., 197376 Saint Petersburg, Russia; kratosloaded@mail.ru (D.A.M.); OAMarkelov@etu.ru (O.A.M.)

**Keywords:** SAW-sensor, SAW-resonator, acceleration measurements, ring-shaped design, stress strained state

## Abstract

We propose the use of aluminum nitride (AlN) membranes acting as sensitive elements for the surface acoustic wave (SAW)-based acceleration measurement. The proposed solution is compared against existing prototypes based on the use of quartz (SiO_2_)/lithium niobate (LiNbO_3_) membranes that are characterized by extensive anisotropic properties. Using COMSOL Multiphysics 5.4 computer simulations we show explicitly that sensitive elements based on less anisotropic AlN membranes overcome both the low sensitivity limitations of SiO_2_ and low temperature stability of LiNbO_3_. Moreover, AlN membranes exhibit nearly double the robustness against irreversible mechanical deformations when compared against SiO_2_, which in turn allows for further 1.5-fold sensitivity enhancement over LiNbO_3_ based sensors. Taking into account their acceptable frequency characteristics, we thus believe that the AlN membranes are a good candidate forsensitive elements especially for high acceleration measurements.

## 1. Introduction

Throughout the 20th century, conventional accelerometer designswere characterized byexcessive weight and size thus preventingthem from widespread utilization. With the advancement of microelectronic technologies sensor sizes could be reduced drastically, while exhibiting considerably lower accuracy and mechanical robustness. The strength of torsions used in the conventional microelectromechanical systems (MEMS) sensorsis strongly limited leading to their inability to withstand overloads caused by excessive acceleration and/or external mechanical forces.

Surface acoustic wave (SAW) based sensors, while less developed to date, provide a reasonable and largely promising alternative. Recent developments based on monolithic solid-state constructionsare characterized by relatively high stability of parameters, low energy consumption (0.5–1 W) [1]. Although SAW-based micromechanical accelerometers (MMA) are currently still under development, commercially available SAW sensors are widely used in other applications ranging from medicine and life safety to unmanned devices exemplified by vapor and gas analyzers [2,3,4], temperature control systems [5,6] as well as pressure detection systems [7].

One of the key requirements for further advancement of SAW based MMAs and similar devices is finding new piezoelectric materials for the sensitive element (SE) console that could overcome typical limitations of the existing prototypes [8,9,10,11].

Very recently [12] we have suggested a SAW-based MMA design based on a ring-type SE to overcome the disadvantages of rectangular [13,14] and triangular shaped SEs [15] due to more even load distribution over the SE surface. In this current study we extend our previous findings towards (i) the optimization of the SE-attachment in the housing, (ii) finding the best material for the perspective SE design according to its frequency characteristics as well as (iii) estimation of the potential impact of the external influences such as excessive acceleration and temperatures on the SE assessed by computer simulations using the COMSOL Multiphysics software package.

## 2. Sensitive Element Design

**Figure 1:**
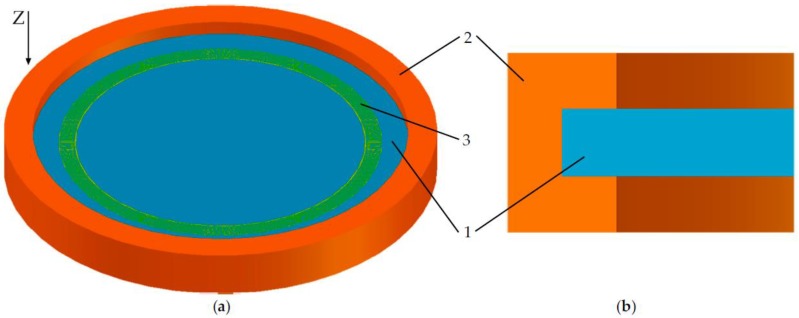
Membrane sensitive element. General view (**a**) and front view (**b**): 1: console; 2: housing; 3: inter-digital transducer.

The overall view of the membrane sensitive element is presented in Figure 1. The model was designed in the AutoCAD 2019 software with the subsequent import of the model into COMSOL Multiphysics 5.4 because of the limited capabilities of the CAD editor of the latter. The resonator consists of two ring-shaped inter-digital transducers (IDT) (1) and piezoelectric crystal located between the transducers (2). The entire structure is limited in both depth and radius by a damping medium to suppress the parasitic reflections of waves from the outer boundaries.

**Figure 2:**
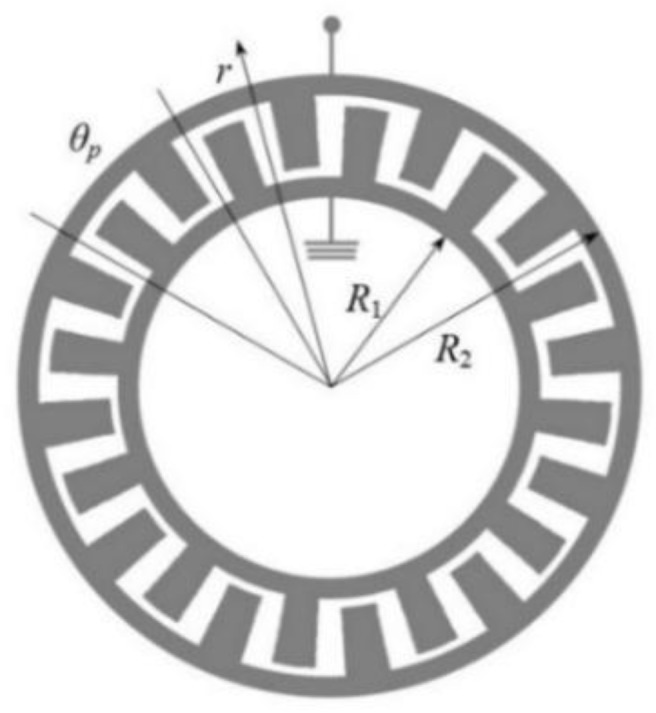
Inter-digital transducer.

The IDT design is shown in Figure 2. The initial overall characteristics of the IDT are taken from the research [16,17]. According to calculations, the IDT period length in the center of the ring is 18.5 µm at an angular period of the transducer equal to *θ_p_* = 1° and height *h* = 0.2 µm. Taking this value for the wavelength and considering that SAWs attenuate at a depth of about three wavelengths, the height of the structure will be 8 wavelengths. It should be noted, that aluminum nitride is a film material and its use as a piezoelectric material requires deposition. AlN deposition is mainly produced on quartz. The quartz thickness for AlN deposition should be at least 3 wavelengths. For convenient results comparing the same overall model characteristics are used, so the total console height will be 12 wavelengths or 222 µm for all materials used in this article. The console radius is 1500 µm.

## 3. Computer Simulation

### 3.1. Sensitive Element Attachment Method

**Figure 3:**
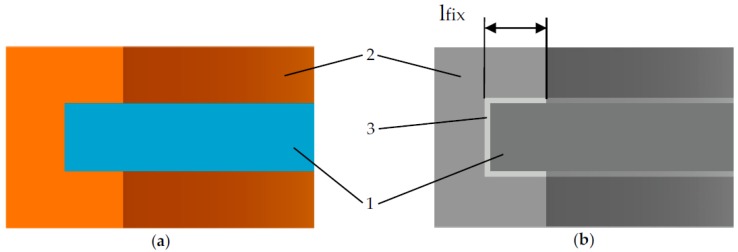
Console attachment methods: rigid (**a**) and using silicone adhesive (**b**): 1: console; 2: housing; 3: silicone adhesive.

**Figure 4:**
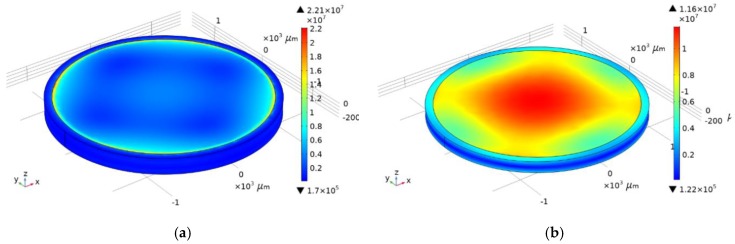
Load distribution for rigid attachment (**a**) and silicone adhesive (**b**).

**Figure 5:**
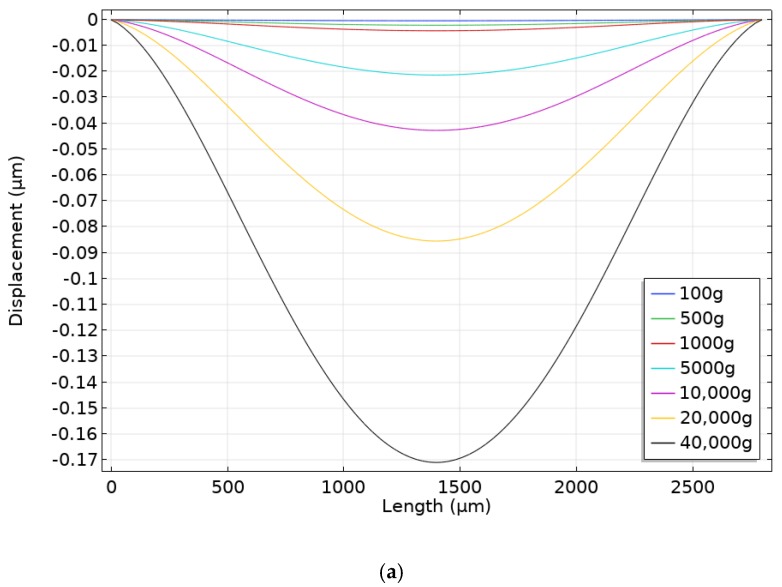

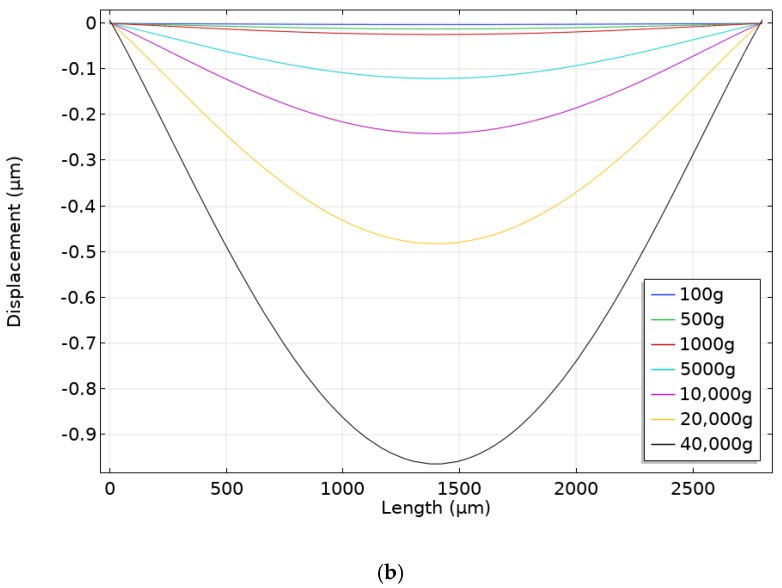
Console displacement with rigid attachment (**a**) and silicon adhesive (**b**).

To determine the optimal way to attach the console to the body and determine the load distribution, it is necessary to create an external influence in the form of acceleration. The characteristics of the materials used are presented in Table 1. The range of acceleration values is 0–40,000 g. Acceleration acts perpendicular to the plane of the console or along the *z* axis (Figure 1). Two models of console attachment were used: with or without silicone adhesive (Figure 3). Console fixed distance in housing (*l*_fix_) was 50 µm. The load distribution and displacement along the diametrical section of the SiO_2_ console at acceleration of 40,000 g are shown in Figure 4 and Figure 5.

Lithium niobate and quartz are anisotropic materials at the same time aluminum nitride is isotropic. Figure 4a,b illustrated that displacements of the material were uneven due to anisotropy when quartz was used as a substrate material. However, in case of aluminum nitride such effect could not be observed and displacements of the material are uniform.

According to the simulation results, when the console is rigidly attached in the sensor housing, the load is concentrated in the attachment area, which will adversely affect the accelerometer sensitivity, since the console experiences a small deformation along the sensitivity axis. When using silicone adhesive to secure the console, the load is evenly distributed over the console area. The optimum distance for the placement of the IDT structure is 1090 µm from the console center. The simulation was carried out for three materials. To demonstrate the effect the sample of four values is presented in Table 2.

According to Table 2, lithium niobate is the most deformed material.

### 3.2. Frequency Characteristic

**Figure 6:**
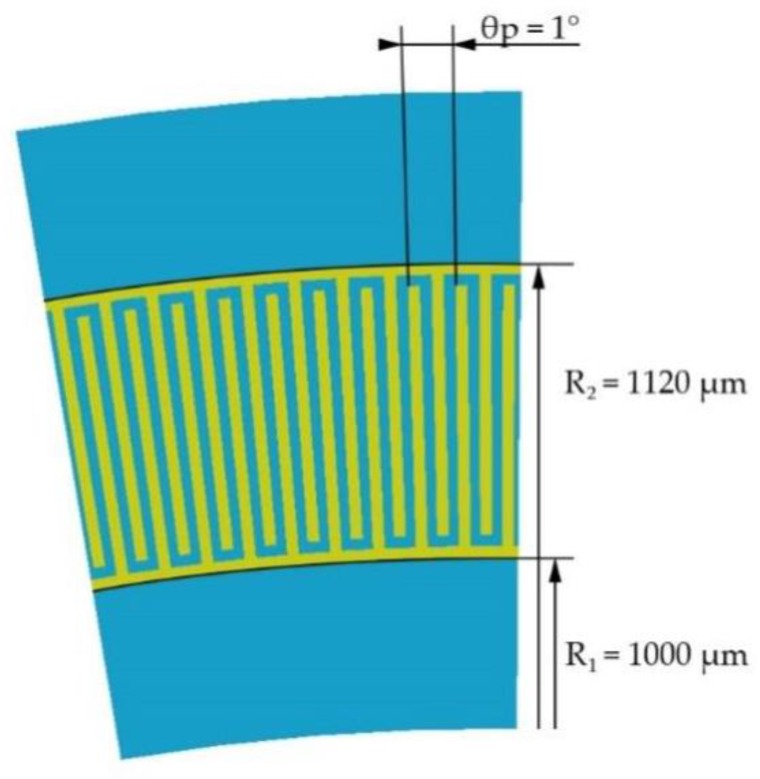
Inter-digital transducer geometry.

To determine the resonance mode for each material, we take the length of one IDT period at 18.5 µm according to the dimensional characteristics of the SE (Figure 6). For the free surface, the resonance frequency for SiO_2_ was 168.21 MHz, LiNbO_3_—212.38 MHz, and AlN—316.49 MHz. It was necessary to take into account that partially metallized or fully metallized surface will reduce the speed of wave propagation on the material surface, which will reduce the resonance frequency. 

**Figure 7:**
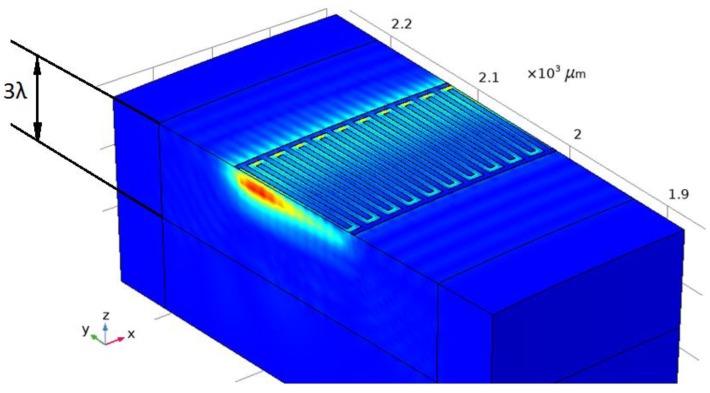
SAW-distribution on console surface.

**Figure 8:**
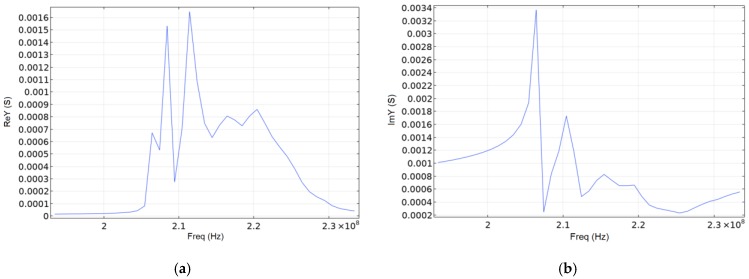
Real (**a**) and imaginary (**b**) admittance component for LiNbO_3_.

**Figure 9:**
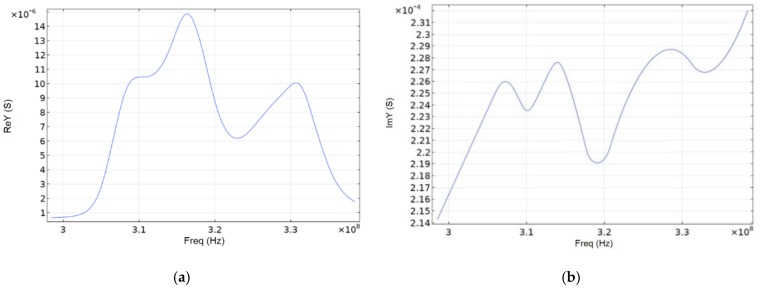
Real (**a**) and imaginary (**b**) admittance component for AlN.

Since the whole SE structure was of the same type, it was possible to use one IDT-period to determine the admittance of materials in the simulation. Figure 7 shows a 5-degree console segment of SE exemplifying the propagation of the SAW for the lithium niobate material. The figure shows that acoustic waves propagated in a coarse distribution up to three wavelengths, which characterizes them as surface acoustic waves. Graphs of the real and imaginary admittance component are shown in Figure 8 and Figure 9.

Comparing Figure 9 and articles [16,17], it can be seen that the peak frequencies diverge by ~10% (286 MHz [16] and 316.5 MHz). From this we can conclude the adequacy of the model used. The frequency difference is due to the fact that over the past 10 years, the piezoelectric characteristics of aluminum have been refined. Also, in our work, only the program features were used. In addition to modeling, the articles [16,17] used the analytical method. We did not consider higher frequencies because it is impractical for SAW based micromechanical sensors due to drastic increase in energy losses and thus also model accuracy in this band is questionable due to the lack of their experimental validation.

**Figure 10:**
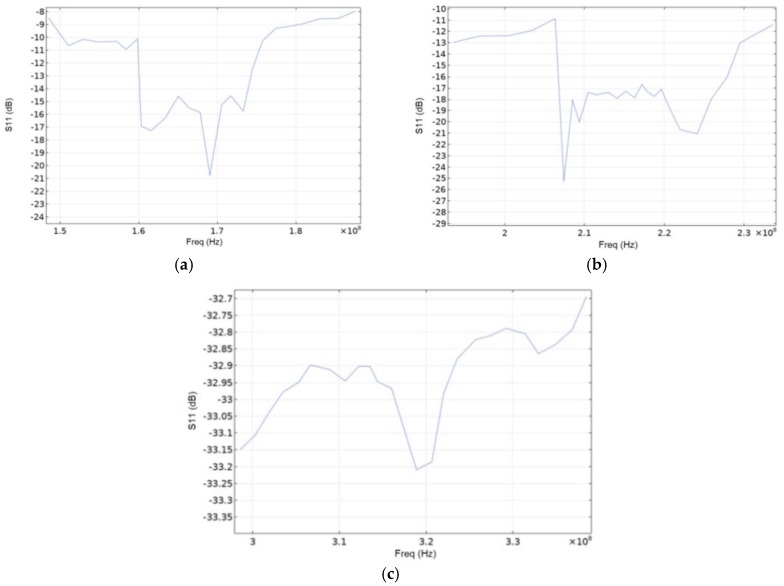
S11 parameter for SiO_2_ (**a**), LiNbO_3_ (**b**) and AlN (**c**).

Figure 10 shows graphs of the S11 parameter for the three console materials. Remarkably, while AlN has largest absolute S11 values, it also exhibits weakest variations of the S11 parameter with frequency drift that could be also advantageous under certain conditions.

The peculiarity of the ring wave resonator on SAW is that the first harmonic, and, consequently, the maximum value of the real component of the admittance, is located on the outer part of the IDT-aperture, and the second—in the central one. According to the simulation results, the highest value of the real component of the complex conductivity for SiO_2_ is 0.168 mS, for LiNbO_3_—88.5 mS, and for AlN—0.887 mS.

**Figure 11:**
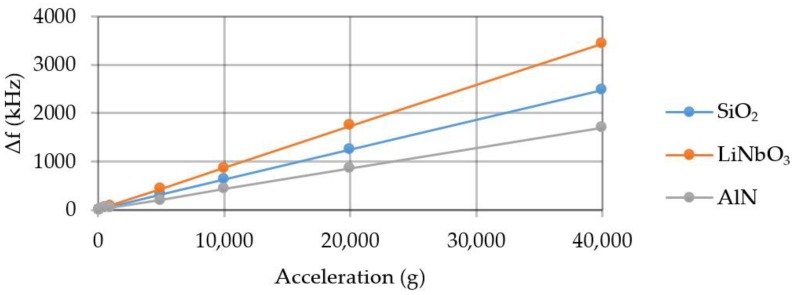
Graph of the frequency change under acceleration.

Possible assessment of the element sensitivity. Figure 11 shows a graph of the frequency change under acceleration in the manufacture of the console from various materials.

The element sensitivity to the forcing acceleration when using SiO_2_ is 65 Hz/g, LiNbO_3_—87 Hz/g, AlN—43 Hz/g in the range up to 40,000 g.

Drawing a conclusion from Figure 7, Figure 8 and Figure 9, we can say that the energy leakage is very significant. Quantitatively, energy leakage depends on the quality factor. The lower the quality factor, the more energy leaves the system. In our case, the model has a quality factor of ~170. This model was used to confirm its adequacy [16,17]. In order to reduce system losses and increase the quality factor, we increased the IDT diameter by two times. The quality factor of the resonator increased by six times, and the energy leakage became insignificant.

### 3.3. External Influences on the SE

**Figure 12:**
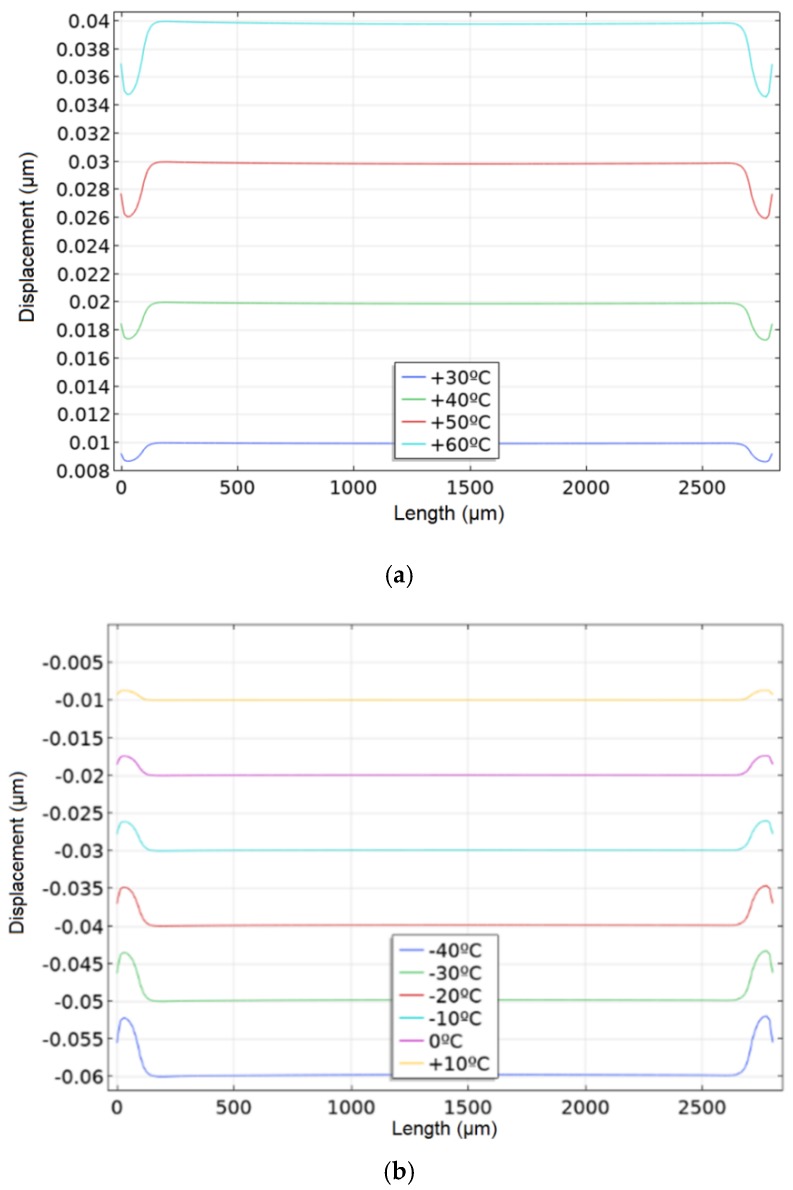
Load distribution (**a**) and displacement (**b**) graphs for quartz.

Temperature has a significant effect on piezoelectric materials. With the help of sensors on SAW it is possible to measure the medium temperature. In this case, the temperature will introduce an error in the measurement of acceleration. To assess its influence and determine the operating temperature range, simulations were performed with values ranging from −40 °C to +60 °C in 5 °C increments for three materials. For example, load distribution and displacement graphs for quartz are shown in Figure 12. Table 3 presents a sample of values to demonstrate the effect.

**Figure 13:**
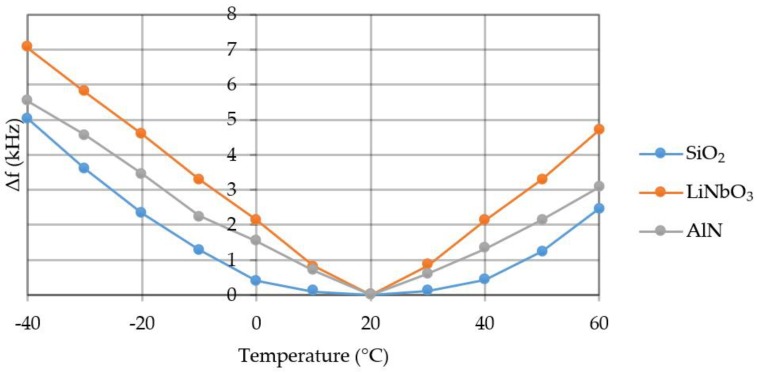
Graph of the frequency change under temperature.

The load is distributed evenly over the console area. The compression or tension of the material comes from the console center. Figure 13 shows a graph of the frequency change under the temperature.

Material, the least susceptible to temperature, is quartz. Its dependence on temperature is in the form of a parabola. The material most exposed to temperature is lithium niobate. Temperature sensitivity when using SiO_2_—~43 Hz/°C, LiNbO_3_—~107 Hz/°C, AlN—~77 Hz/°C in the range of −40 °C to 60 °C.

**Figure 14:**
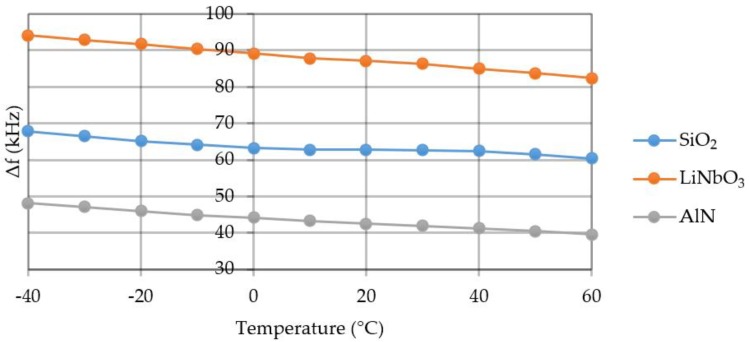
Graph of the frequency change depending on the temperature at an acceleration of 100 g.

Simulation of the mutual influence of acceleration and temperature on the SE was conducted in the ranges specified in Section 3.1 and Section 3.3. For example, Table 4 shows a sample of the simulation results for quartz, and Figure 14 shows a graph of the frequency change depending on the temperature at an acceleration of 100 g.

Based on the data obtained, it can be seen that the introduced error, depending on the compression or expansion of the material, is added or subtracted, respectively, from the value of the frequency change at the current acceleration and T = 20 °C. 

**Figure 15:**
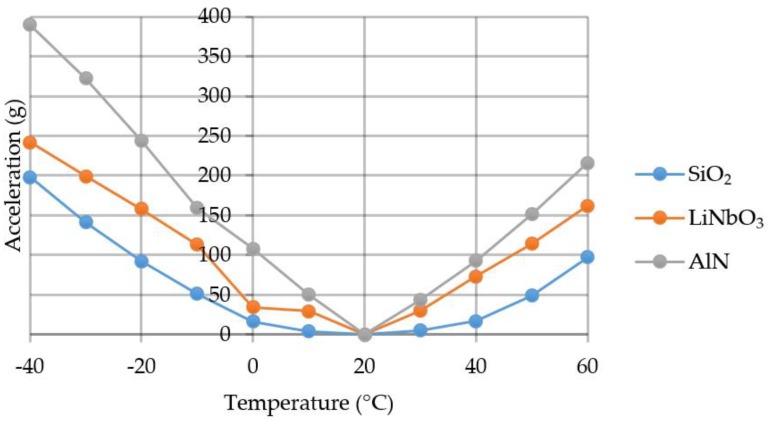
Graph of the minimum acceleration value.

If we consider that the useful signal should be 3 times higher than the noise, the measurements are possible with a certain acceleration value. The graph of the minimum defined acceleration value is shown in Figure 15.

The average minimum defined acceleration for lithium niobate was 3.5 g/°C, for aluminum nitride −5.4 g/°C, and for quartz −1.7 g/°C. The use of aluminum nitride as a SE-material is therefore might be not the best choice especially in those applications where significant temperature variations can be observed.

### 3.4. Placing the Inertial Mass on the Console

To increase the sensitivity of the device, which is a consequence of increasing the console deformation, it is possible to place the inertial mass (IM) in the form of a cylinder in the console center. Based on the data from Section 3.1 and Section 3.3, the quartz is experiencing tension close to the tensile strength, lithium niobate, and aluminum nitride have two-fold and four-fold safety margins, respectively, with the highest values of external influences (40,000 g and −40 °C). 

According to this, for quartz placement of IM will be accompanied by a decrease in the investigated ranges affecting SE, accelerations or temperatures. It is possible to simulate all the investigated external influences for other materials used in the work. 

**Figure 16:**
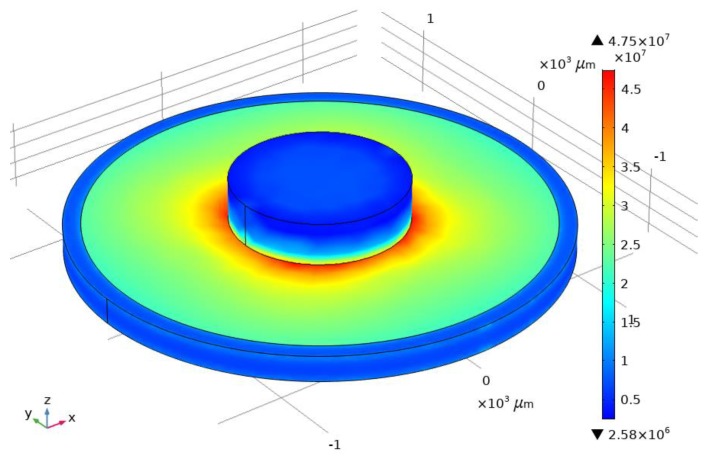
Load distribution in the presence of IM.

**Figure 17:**
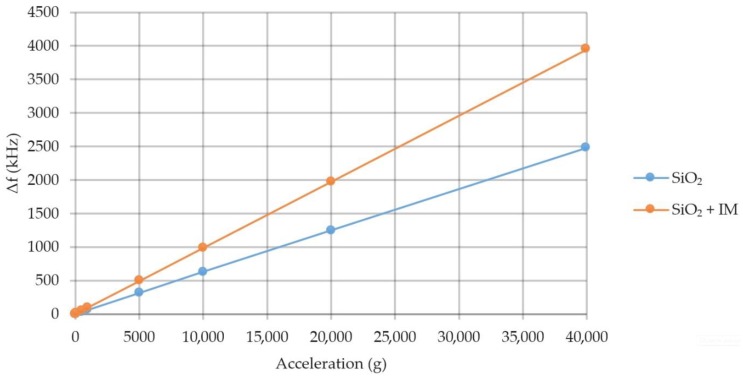
Graph of the frequency change under acceleration.

For example, a quartz cylinder with a volume of 0.049 mm^3^ was placed on the quartz console. Simulation of the influence of acceleration and temperature was conducted on the values specified in Section 3.1 and Section 3.3. The voltage field of the SE is shown in Figure 16 and Figure 17 shows a graph of the sensitivity of the device with IM placed on it.

According to the simulation results, it can be concluded that quartz was destroyed at the values of the forcing acceleration above 20,000 g at a temperature of 60 °C. The device sensitivity in the absence of exposure to temperature increased from 65 Hz/g to 86 Hz/g.

### 3.5. Overall Design Recommendations

Based on our results specified above, we recommend the following design and specifications:

(i) The console should be fixed by its attachment to the housing at 50 µm distance from its center using silicone adhesive;
(ii) The SAWs should be located at 1090 µm from the console center;
(iii) The SE sensitivity to the acceleration is approximately 65 Hz/g for the SiO_2_, 87 Hz/g for the LiNbO_3_, and 43 Hz/g for the AlN for the accelerations up to 40,000 g.
(iv) The console should be preferentially manufactured from the lithium niobate YX128°-cut as it exhibits more pronounced frequency variations under similar accelerations being applied;
(v) The temperature sensitivity when is approximately ~43 Hz/°C for the SiO_2_, ~107 Hz/°C for the LiNbO_3_, ~77 Hz/°C for the AlN at least within the studied range between −40 °C to 60 °C.
(vi) The use of aluminum nitride as a SE-material is limited due to its lower temperature stability especially when pronounced temperature variations are expected;
(vii) To further enhance the sensitivity, it is advised to place the IM in the center of the console, although one should take into account that the presence of IM will reduce the range of measurements.

## 4. Conclusions

To summarize, we have proposed the use of AlN membranes acting as sensitive elements for the SAW-based acceleration measurement. The proposed solution has been compared against existing prototypes based on the use of SiO_2_/LiNbO_3_ membranes that are characterized by extensive anisotropic properties. Using COMSOL Multiphysics computer simulations we have shown explicitly that sensitive elements based on less anisotropic AlN membranes overcome both the low sensitivity limitations of SiO_2_ and low temperature stability of LiNbO_3_. Moreover, AlN membranes exhibits nearly double robustness against irreversible mechanical deformations when compared against SiO_2_, which in turn allows for further 1.5-fold sensitivity enhancement over the LiNbO_3_ based sensors. Taking into account their acceptable frequency characteristics, we thus believe that the AlN membranes are perspective sensitive elements especially for high acceleration measurements.

## Figures and Tables

**Table 1:** Characteristics of piezoelectric materials and silicone adhesive.

Parameter	ST-Cut SiO_2_	YX-128°-Cut LiNbO_3_	AlN	Silicone Adhesive
Wave velocity, *v_p_* [m/s]	3158	3961	5705	-
Density, *ρ* [kg/m^3^]	2650	4640	3300	1700
Elastic modulus, *E* [Pa]	76.5 × 10^9^	170 × 10^9^	310 × 10^9^	25 × 10^6^
Poisson’s ratio, *v*	0.08	0.25	0.24	0.48
Tensile strength, *F* [Pa]	48 × 10^6^	110 × 10^6^	250 × 10^6^	-
Thermal Expansion, *α* [K^−1^]	13.37 × 10^−6^	14.8 × 10^−6^	5.6 × 10^−6^	275 × 10^−^^6^
Thermal Conductivity, *λ* [W/m·K]	6.5	4.6	170	1.375
Specific Heat, *c* [J/kg·K]	744	630	780	1175

**Table 2:** Deformation of the console when using silicone adhesive (µm).

Acceleration, g	SiO_2_	LiNbO_3_	AlN
50	0.0013059	0.0013131	0.0004800
1000	0.0261190	0.0262620	0.0094005
40,000	0.9847500	1.0505000	0.3880200

**Table 3:** Deformation of the console when heating or cooling (µm).

Temperature, °C	SiO_2_	LiNbO_3_	AlN
−40	−0.061019	−0.078018	−0.027821
−20	−0.040874	−0.052467	−0.018502
0	−0.020364	−0.027518	−0.009658
+20	0	0	0
+40	0.020326	0.027506	0.009543
+60	0.040852	0.052125	0.018602

**Table 4:** Deformation of the console when heating or cooling and acceleration (µm).

Temperature, °C	50 g	500 g	5000 g	40,000 g
−40	−0.062	−0.072	−0.180	−1.024
−10	−0.031	−0.042	−0.150	−0.994
+10	−0.011	−0.022	−0.130	−0.975
+40	0.021	0.032	0.140	0.985
+60	0.042	0.052	0.160	1.004
